# Structural, electronic, vibration and elastic properties of the layered AgInP_2_S_6_ semiconducting crystal – DFT approach

**DOI:** 10.1039/c7ra13519j

**Published:** 2018-02-13

**Authors:** T. Babuka, K. Glukhov, Y. Vysochanskii, M. Makowska-Janusik

**Affiliations:** Institute of Physics, Faculty of Mathematics and Natural Science, Jan Dlugosz University in Czestochowa Al. Armii Krajowej 13/15 42200 Czestochowa Poland m.makowska@ajd.czest.pl; Institute for Solid State Physics and Chemistry, Uzhgorod National University 54 Voloshyn St. 88000 Uzhgorod Ukraine

## Abstract

Detailed first principles calculations of the structural, electronic and vibrational properties of the AgInP_2_S_6_ crystal are reported. The energy band spectra of the mentioned material using DFT/GGA-D methodology with the PBE functional was calculated for the first time. Stability of the AgInP_2_S_6_ crystals in contrast to Cu-containing representatives of M1M2P_2_X_6_ materials family (M1, M2 – metal, X – chalcogen) has been explained in the framework of the second-order Jahn–Teller effect. The high covalence of the Ag–[P_2_S_6_] bonds and strong hybridization of the 4d- and 5s-orbitals of the Ag atoms are responsible for the stability of the considered crystal. The calculated vibrational properties were compared with the available experimental data derived from Raman scattering spectroscopy and their good agreement was demonstrated. The electronic and vibration properties within the framework of a group theory approach were studied. Also elastic properties of the AgInP_2_S_6_ crystal were modeled and analyzed for the first time.

## Abstract 2

First principles calculations of the structural, electronic and vibrational properties of the AgInP_2_S_6_ crystal were reported. Data were analyzed within the framework of the group theory approach. Results were compared to the experimental data.

## Graphical abstract

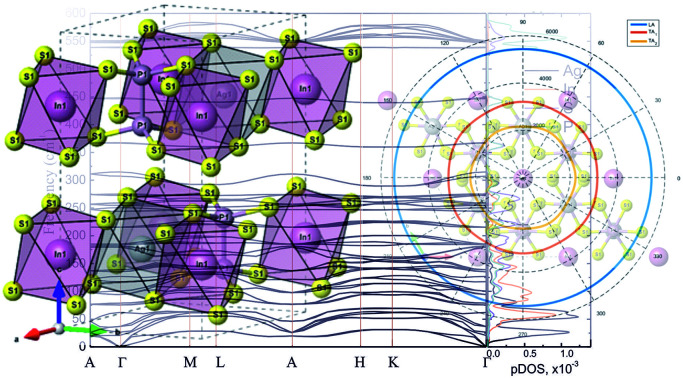

## 1 Introduction

Recently, hexachalcogenohypodiphosphates have attracted great attention due to their unique physical and chemical properties. Layered crystals belonging to the M1M2P_2_X_6_ (M1, M2 – metal cations, X – chalcogen) family are promising materials for functional electronics, because many of them exhibit ferroelectric, ferro- or antiferromagnetic as well as piezoelectric properties.^1^ They exhibit mixed electron–ionic conductivity^2–4^ and promising optical^5,6^ and thermoelectric properties.^7^ Therefore, these layered crystals are interesting from the fundamental point of view and also they are important materials for practical as well as for technological applications. In this context, it is important to explain the microscopic origin of the wide variety of above-mentioned properties and their dependence on morphological and componential peculiarities of particular representatives of the M1M2P_2_X_6_ crystal family.

Recently, an exploration of functional 2D materials, besides graphene, evolved rapidly thanks to the synthesis of the variety of new materials and their functionalities.^8–17^ In the recent works^8–23^ the main interest is primarily focused on the unique electronic and optical properties of the bulk metal phosphorus trichalcogenides. Especially it applies to the quaternary compounds belonging to the M_1/2_^I^M_1/2_^III^PX_3_ type crystals (mainly M_1/2_^I^ = Cu, Ag; M^III^ = Cr, V, Al, Ga, In, Bi, Sc, Er, and Tm; X = S and Se).^24^ In addition, the nonlinear effects as second harmonic generation (SHG) and chiral electroluminescence appearing in thin atomic layers have expanded the functionalities and potential applications of the 2D materials. The mentioned effects disappear in the centrosymmetric bulk counterparts.^25–30^ Also, the broken inversion symmetry in monolayer 2D materials has shown the viability of optical valley control.^31,32^ Furthermore, the external modulation opens a path to explore the physical and technological applications of the functional 2D materials. One of the most important result that was obtained in recent years is the fact that the CuInP_2_S_6_ ferrielectric is a promising functional material with polarization switching not only in bulk samples but even in thin flakes of few tens nanometers thickness.^33,34^

Consideration of materials with different chemical diversity and structural complexity gives the possibility to investigate their comprehensive properties. Recently, the creation of artificial materials (heterostructures) based on components with the same structural properties is very popular. The heterostructure constructed from ferrielectric CuInP_2_S_6_ and paraelectric In_4/3_P_2_S_6_ crystals may be here mentioned.^35^ Semiconducting AgInP_2_S_6_ crystal discussed in present work also have a similar structure. Thus, one can assume the possibility to create an artificial materials on its basis. However, application of the AgInP_2_S_6_ crystal in the future, first of all needs their physical properties investigations.

The AgInP_2_S_6_ crystal was chosen as material representing the M1M2P_2_X_6_ family. For the first time, the mentioned semiconductor was synthesized in 1987 and its crystalline structure was reported in the work of Ouili and co-workers.^36^ Being isostructural with the paraelectric phase of the CuInP_2_Se_6_ crystal, the AgInP_2_S_6_ structure does not manifest the presence of the dipole ordering and the ferroelectric phase transition.^6^ Detailed microscopic investigations of an origin of the AgInP_2_S_6_ crystal stability can give also a valuable insight to explain an intriguing behavior of other representatives of the above-mentioned crystal family. Also, the stable atomic thin flacks offers a platform to manipulate the structure of an artificial magnetic or ferroelectric samples by their doping using relevant atoms. Obviously, a synthesis condition of such new functional materials could be elaborated only based on known and clearly described physical properties of the host crystal.

In this case, the present investigations are focused on physical properties modeling of the AgInP_2_S_6_ crystal using quantum chemical methods. To our knowledge, information about properties of the pure AgInP_2_S_6_ crystals are almost absent in the literature. All these facts motivated us to study the mentioned unexplored system. We present the quantum chemical calculation of the structural, electronic, vibrational and elastic properties of the AgInP_2_S_6_ crystals, their analysis, and comparison with experimental data.

The presented paper is organized as follows: in the next section, the structure of the AgInP_2_S_6_ crystal accompanied by symmetry considerations and method of physical properties calculations are described. After that, the paper continues with presenting the results of the structural, electronic, vibrational and elastic properties calculations accompanied by their analysis. The work is ended by conclusions with a short summary.

## 2 Crystal structure of the AgInP_2_S_6_ crystal and method of calculations

**Fig. 1:**
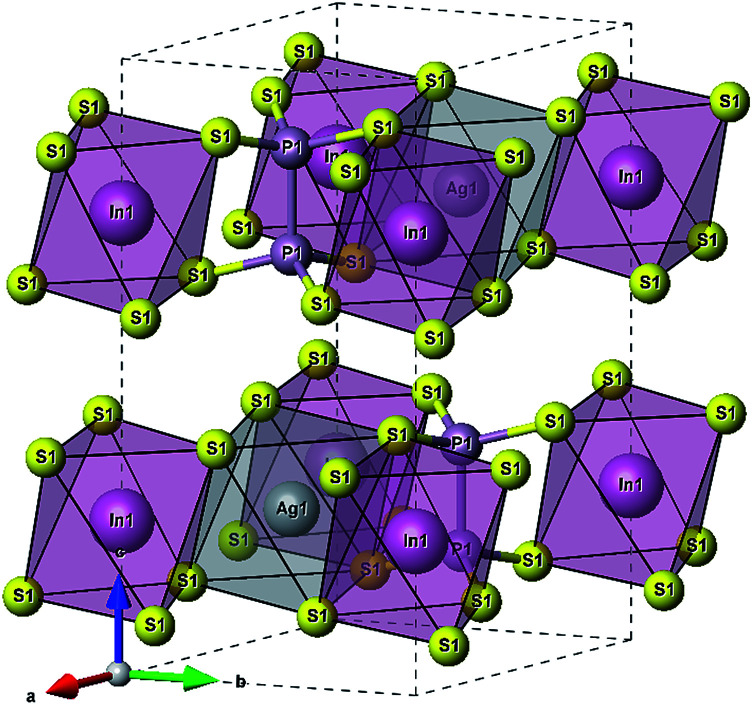
The unit cell of the AgInP_2_S_6_ crystal structure and topology of the Ag- and In-centered polyhedrons.

According to ref. 36, the AgInP_2_S_6_ crystallizes in rhombohedral structure with the *P*3̄1*c* space group (no. 163). The lattice parameters and atomic reduced coordinates are collected in the Table 1. The AgInP_2_S_6_ crystal has prominent layered structure with two nonequivalent packet layers in the unit cell. They may be superimposed by the 180 degrees rotation around the Oz crystallographic axis and are separated by the so-called van der Waals (vdW) gap. Each packet layer consists of the [P_2_S_6_] anionic complex and two metallic cations (Ag and In) located at the center of sulfur near-octahedral polyhedrons connected one with the other by edges (Fig. 1). Every Ag-centered polyhedron is connected with three In-centered polyhedrons (and *vice versa*) meeting the threefold rotational symmetry of the *P*3̄1*c* space group.

**Table 1:** Atomic positions in the hexagonal AgInP_2_S_6_ crystal (reduced coordinates)

Atoms	*x*	*y*	*z*	Wyckoff position	Site symm.
Ag	2/3	1/3	1/4	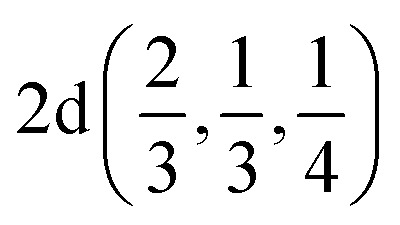	3.2
In	0.0	0.0	1/4	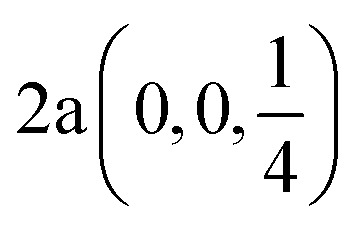	3.2
P	1/3	2/3	0.16376	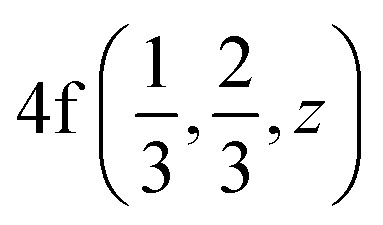	3
S	0.32303	0.34647	0.11906	12i(*x*, *y*, *z*)	1
*a* [Å]	6.18200				
*b* [Å]	6.18200				
*c* [Å]	12.9570				
*V* [Å^3^]	428.837				

**Fig. 2:**
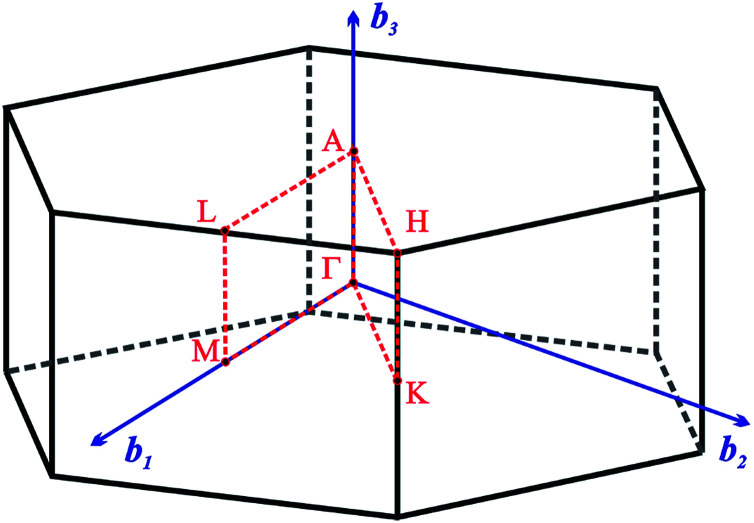
The hexagonal Brillouin zone and *k*-path used for electron and phonon spectra calculations. Coordinates of the high-symmetry points are the following: *Γ*(0, 0, 0), 
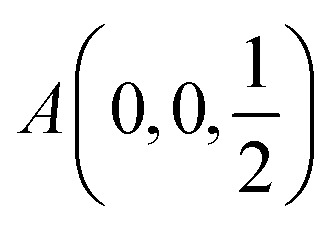
, 
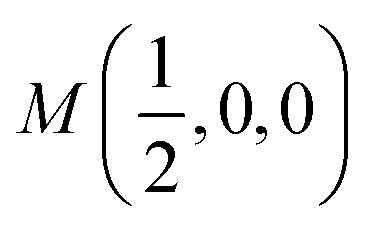
, 
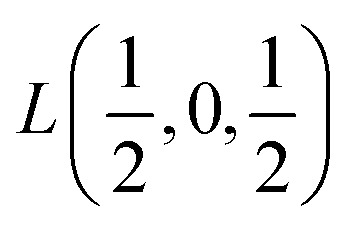
, 
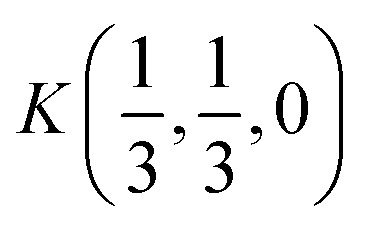
, and 
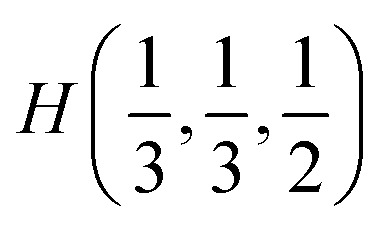
.

The Fig. 2 presents *k*-path involving a set of high-symmetry points in the hexagonal Brillouin zone (BZ) used to calculate the electronic and phonon properties of the AgInP_2_S_6_ crystal structure. Additionally, as an initial input for all performed calculations of the structural, electronic and vibrational properties of the mentioned crystal the structural data from ref. 36 were taken. The CASTEP, module^37^ of the Materials Studio program package, was used to carry out planned calculations. They were performed applying the generalized gradient approximation (GGA) with the Perdew–Burke–Ernzerhof functional^38^ or with the CA-PZ local functional based on the Ceperley and Alder data^39^ parameterized by Perdew and Zunger.^40^ The AgInP_2_S_6_ crystal possesses layered structure characterized by the presence of so-called vdW gap. Therefore, the extended DFT methodology is needed to calculate electronic properties of the mentioned material. In this case, one may propose to use the DFT-D method taking into account dispersion interaction elaborated by Grimme.^41^ The ultra-soft pseudopotential^42^ was used to perform calculations for Ag – 4d^10^5s^1^5p^0^, In – 4d^10^5s^2^5p^1^, P – 3s^2^3p^3^, S – 3s^2^3p^4^ atomic configurations.

The plane-wave basis set cut-off was chosen to be equal to 600 eV. The Monkhorst–Pack *k*-points grid^43^ sampling was set at 12 × 12 × 3 points for the Brillouin zone. The convergence tolerance parameters were as follows: energy 5 × 10^−6^ eV, force 0.01 eV Å^−1^; stress 0.02 GPa; displacement 0.05 Å. The total energy convergence criterion was assumed to be fulfilled when the self-consistent field (SCF) tolerance reach the value 10^−7^ eV per atom.

## 3 Results of calculations

### 3.1. Structural properties and symmetry analysis

As it was mentioned above, symmetry of the AgInP_2_S_6_ crystal lattice is defined by the *P*3̄1*c* (*D*_3d_^2^) space group. Symmetry operations 
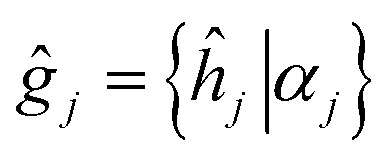
 (*h*_*j*_ – rotational part, *α*_*j*_ – nontrivial translation, 
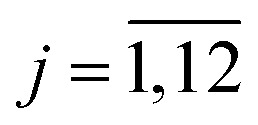
) suitable for mentioned structure are as follows:1
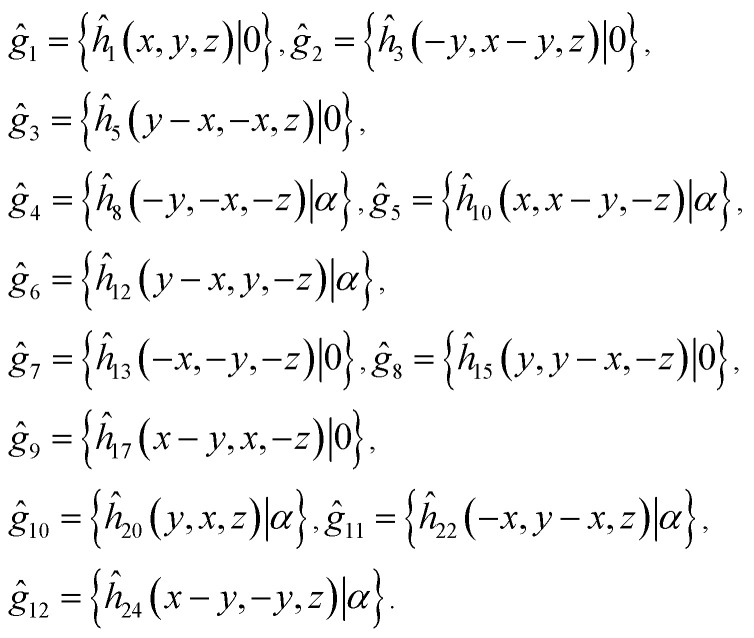


Following the rules contained in the book of Kovalev^44^ the possible non-trivial translations in relative coordinates 
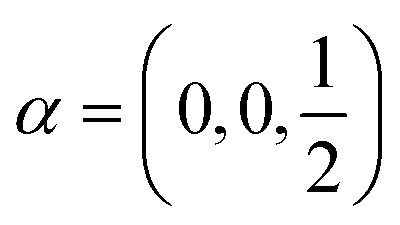
 and rotations denoted in the oblique coordinate system can be classified into six classes:2
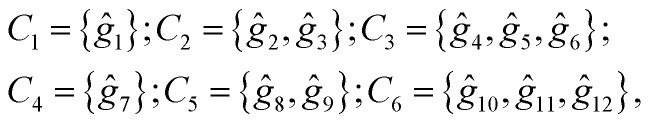
corresponding to the identity operation (E), two threefold rotations (C_3_), three horizontal twofold screw rotations (C′_2_), inversion (I), inversion rotations (S_3_) and vertical glide planes (m_v_), respectively. Table of characters of the irreducible representation (irrep) of little groups of the *D*_3d_^2^ group in high-symmetry points in the BZ and corresponding basis functions are presented in Table 2. The characters of the irreps presented in Table 2 can be used to establish the selection rules for the direct dipole optical transition for example in the center of BZ. It is well known that matrix element of the dipole transition is nonzero when the direct product of irreps Γ^*^_α_ × *Γ*_*β*_ × *Γ*_*γ*_ contains the *Γ*_1_ irrep. In the mentioned expressions the *Γ*_*α*_ corresponds to the irrep of the initial state, *Γ*_*γ*_ describes the symmetry of a final state and *Γ*_*β*_ is an irrep describing transformation rules of a radius-vector *r⃑*(*x*, *y*, *z*) which coincides with a dipole operator. One may assume that the initial state has *Γ*_6_ symmetry, final state transforms according to *Γ*_1_ and the polarization of incident light is in *XOY* plane (symmetry is *Γ*_6_). Consequently, the direct product is as follows Γ^*^_6_ × *Γ*_6_ × *Γ*_1_ = *Γ*_1_ + *Γ*_3_ + *Γ*_5_. As far as the product contains *Γ*_1_ the above transition is allowed. Summary of all possible initial and final states and all possible polarizations is presented in Table 3. In particular, the direct *Γ*_6_ → *Γ*_1_ transition is allowed only for *E* ⊥ OZ polarization.

**Table 2:** Characters of irreps and the basis functions of little groups of the *D*_3d_^2^ space group in high-symmetry points of BZ. Here (*x*, *y*, *z*) and (*J*_*x*_, *J*_*y*_, *J*_*z*_) are components of polar and axial vectors, respectively. Representations in curly brackets are combined due to the time inversion symmetry. Shaded cells represent the symmetry operations absent in corresponding little groups

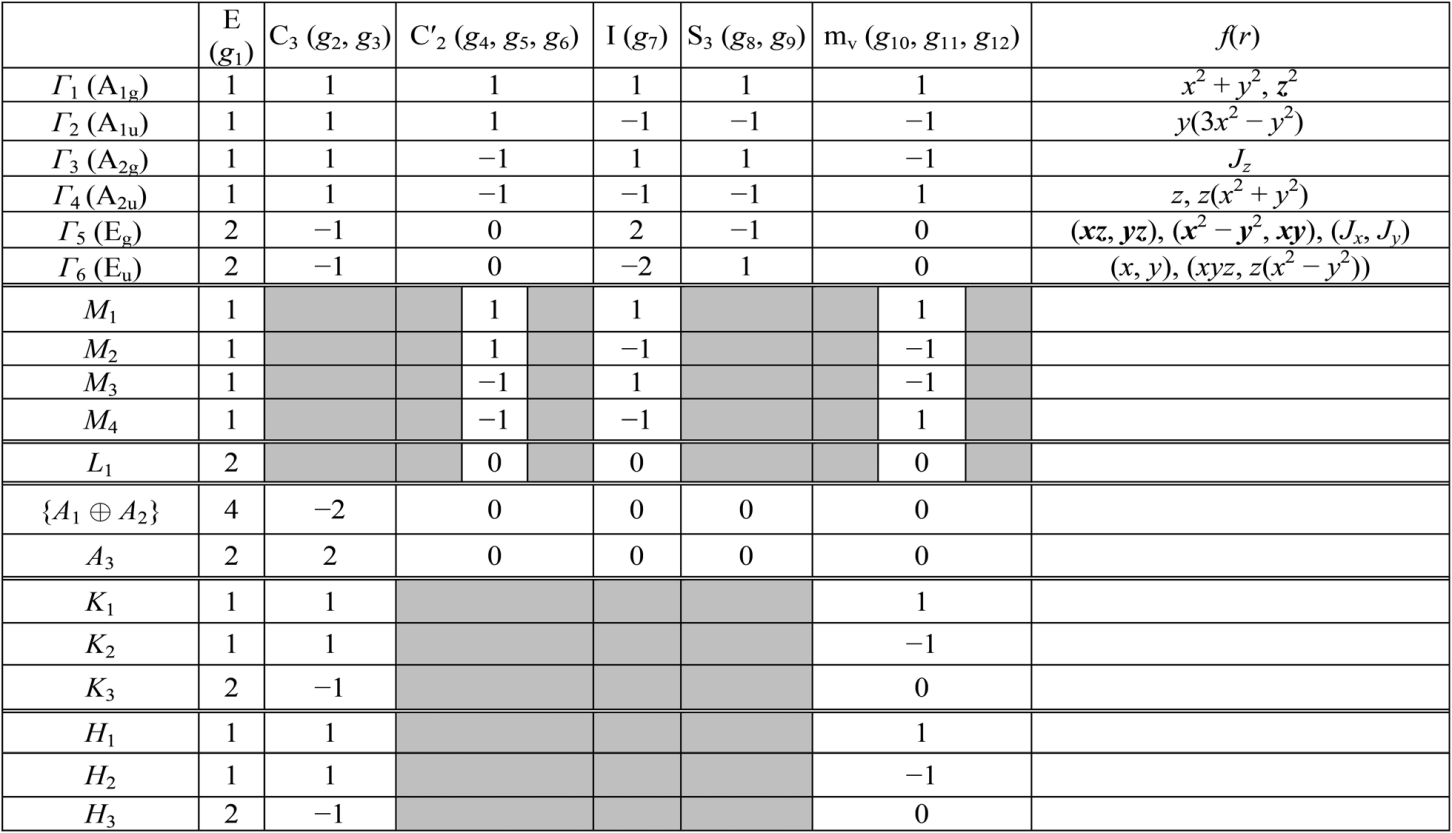

**Table 3:** Summary of all possible initial and final states and all possible polarizations

Initial state	Allowed final states	
	*E* ‖ OZ (*Γ*_4_)	*E* ⊥ OZ (*Γ*_6_)
*Γ*_1_	*Γ*_4_	*Γ*_6_
*Γ*_2_	*Γ*_3_	*Γ*_5_
*Γ*_3_	*Γ*_2_	*Γ*_6_
*Γ*_4_	*Γ*_1_	*Γ*_5_
*Γ*_5_	*Γ*_6_	*Γ*_2_, *Γ*_4_, *Γ*_6_
*Γ*_6_	*Γ*_5_	*Γ*_1_, *Γ*_3_, *Γ*_5_

As it was mentioned above, the AgInP_2_S_6_ crystal structure has two nonequivalent layers in the unit cell. In the quasiparticle spectra of such crystals the small splitting, known as Davydov's splitting, take place in dispersion curves.^45^ Group theory allows to analyze these states basing on the knowledge of the single layer symmetry. In the considered structure, the symmetry group of the single layer is *D*_3_^1^ and it contains the following six operations:3
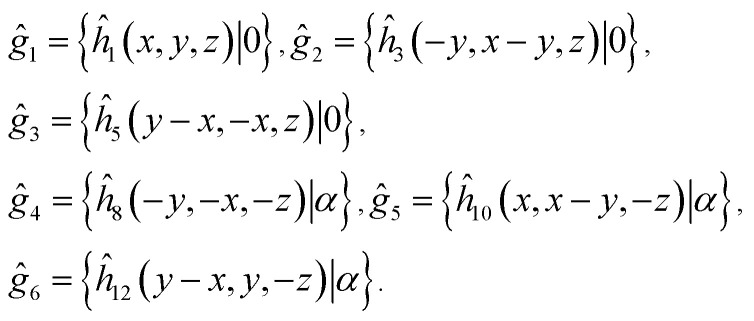


Irreducible representations of the mentioned subgroup in the center of the BZ are collected in Table 4. Symmetry analysis allows to establish relations between irreducible representations of the *D*_3d_^2^ group and its subgroup *D*_3_^1^ giving the symmetry description of Davydov's splitting. Results of this relations are summarized in the following diagrams:

4 *Γ*^layer^_1_ → (*Γ*_1_, *Γ*_2_); *Γ*^layer^_2_ → (*Γ*_3_, *Γ*_4_); *Γ*^layer^_3_ → (*Γ*_5_, *Γ*_6_).

**Table 4:** Characters of irreps of the symmetry group corresponding to a single layer of AgInP_2_S_6_

	E (*g*_1)_	C_3_ (*g*_2_, *g*_3_)	C′_2_ (*g*_4_, *g*_5_, *g*_6_)	*f*(*r*)
*Γ* ^layer^_1_ (A_1_)	1	1	1	1, *x*^2^ + *y*^2^, *z*^2^
*Γ* ^layer^_2_ (A_2_)	1	1	−1	*z*, *J*_*z*_
*Γ* ^layer^_3_ (E)	2	−1	0	(*x*, *y*), (*xz*, *yz*), (*x*^2^ − *y*^2^, *xy*), (*J*_*x*_, *J*_*y*_)

The above relations mean that all branches of the AgInP_2_S_6_ crystal band spectrum will be the symmetrically split doublets defining transformation rules by above-written couples of irreps.

Knowing the site symmetry of atomic positions (see Table 1) and thus the localization of the valence charge the energy band structure can be predicted by elementary energy bands concept.^46,47^ Using the induction procedure^48,49^ the representations of the crystal space group induced by irreps of the site symmetry groups for particular Wyckoff positions could be obtained. These induced representations are the elementary energy bands building the whole valence band. The structure of these elementary energy bands originating from atomic positions are present below:

position 
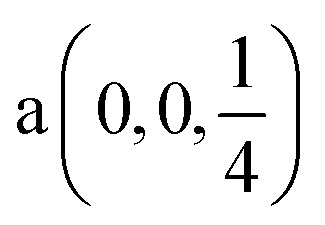


A_1_: *Γ*_1_ + *Γ*_2_ − *M*_1_ + *M*_2_ − *L*_1_ − *A*_3_ − *K*_1_ + *K*_2_ − *H*_1_ + *H*_2_;

A_2_: *Γ*_3_ + *Γ*_4_ − *M*_3_ + *M*_4_ − *L*_1_ − *A*_3_ − *K*_1_ + *K*_2_ − *H*_1_ + *H*_2_;

E: *Γ*_5_ + *Γ*_6_ − *M*_1_ + *M*_2_ + *M*_3_ + *M*_4_ − 2*L*_1_ − {*A*_1_ ⊕ *A*_2_} − 2*K*_3_ − 2*H*_3_;

position 
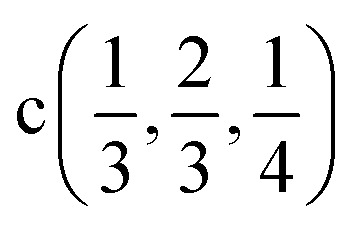
 and position 
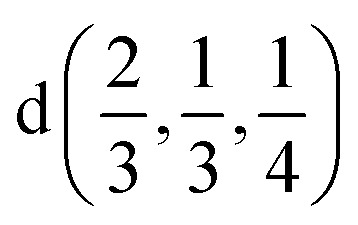


A_1_: *Γ*_1_ + *Γ*_2_ − *M*_1_ + *M*_2_ − *L*_1_ − *A*_3_ − *K*_3_ − *H*_3_;

A_2_: *Γ*_3_ + *Γ*_4_ − *M*_3_ + *M*_4_ − *L*_1_ − *A*_3_ − *K*_3_ − *H*_3_;

E: *Γ*_5_ + *Γ*_6_ − *M*_1_ + *M*_2_ + *M*_3_ + *M*_4_ − 2*L*_1_ − {*A*_1_ ⊕ *A*_2_} − *K*_1_ + *K*_2_ + *K*_3_ − *H*_1_ + *H*_2_ + *H*_3_;

position 
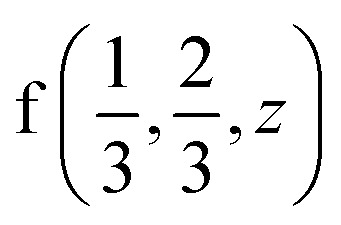


A_1_: *Γ*_1_ + *Γ*_2_ + *Γ*_3_ + *Γ*_4_ − *M*_1_ + *M*_2_ + *M*_3_ + *M*_4_ − 2*L*_1_ − 2*A*_3_ − 2*K*_3_ − 2*H*_3_;

E: *Γ*_5_ + *Γ*_6_ − *M*_1_ + *M*_2_ + *M*_3_ + *M*_4_ − 2*L*_1_ − {*A*_1_ ⊕ *A*_2_} − *K*_1_ + *K*_2_ + *K*_3_ − *H*_1_ + *H*_2_ + *H*_3_.

For general position i(*x*, *y*, *z*) all corresponding irreps are present in each *k*-point:

A: *Γ*_1_ + *Γ*_2_ + *Γ*_3_ + *Γ*_4_ + 2*Γ*_5_ + 2*Γ*_6_ − 3(*M*_1_ + *M*_2_ + *M*_3_ + *M*_4_) − 6*L*_1_ − 2({*A*_1_ ⊕ *A*_2_} + *A*_3_) − 2(*K*_1_ + *K*_2_ + 2*K*_3_) − 2(*H*_1_ + *H*_2_ + 2*H*_3_).

The induced representations for position 
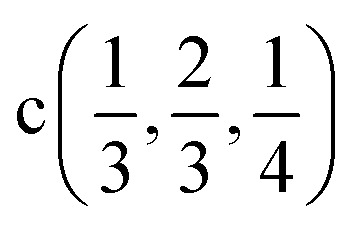
 located in the middle of P–P bond are also listed above. Their structure completely coincides with those induced by irreps for the local position 
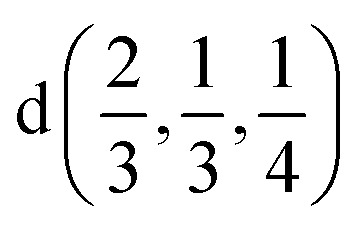
. These relations will be used to analyze the structure of the calculated energy band spectrum and to investigate the spatial valence charge density distribution depending on the symmetry of the structure.

The electronic properties calculations were performed for fully optimized AgInP_2_S_6_ crystal structure. It means that the atomic positions and lattice parameters were relaxed according to the total energy minimization. The optimization was performed using the GGA/PBE and GGA/PBE-D methodology. It turned out that the lattice parameters obtained by using the GGA/PBE methodology are somewhat greater than the experimental ones. The data obtained using the GGA/PBE-D method are better matched to the experimental results. These results are collected in Table 5. Good compatibility of experimental data with theoretical ones allows to conclude that the applied calculation methodology can be used to predict an electronic and vibrational properties of the AgInP_2_S_6_ crystal presented in the next sections.

**Table 5:** Experimental and calculated structural parameters obtained by GGA/PBE-D methodology for hexagonal AgInP_2_S_6_ crystal

Bond identification	Bond length	
	Exp.^34^ [Å]	GGA/PBE-D [Å]
Ag1–S1	2.7784(10)	2.74837
Ag1–In1	3.5692(70)	3.55313
Ag1–P1	3.7387(80)	3.72616
In1–P1	3.7387(80)	3.72616
P1–P1	2.2260(19)	2.24459
In1–In1	6.1820(11)	6.15420
In1–S1	2.6457(90)	2.67644
S1–S1	3.3715(15)	3.36013
Ag1–Ag1	7.3966(10)	7.41372
*a* = *b*	6.182(2)	6.154207
*c*	12.957(2)	13.013575
*V*, [Å^3^]	428.8(4)	426.846

### 3.2. Electronic properties

**Fig. 3:**
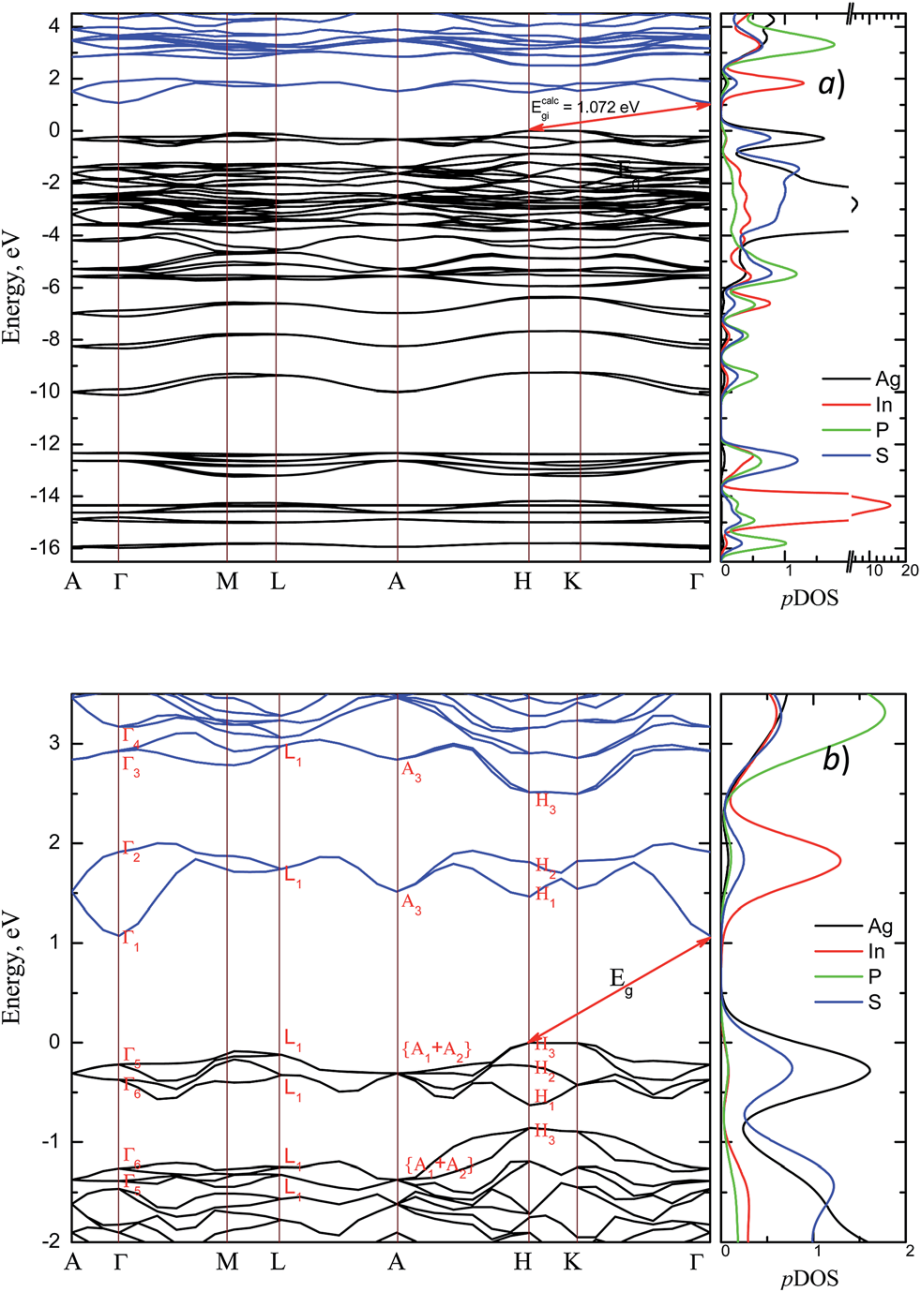
Energy band structure of the AgInP_2_S_6_ crystal and corresponding partial DOS (pDOS) calculated by GGA/PBE-D methodology: overall view (a) and the vicinity of the band gap (b).

The energy band structure and the partial density of states (pDOS) calculated by GGA/PBE-D methodology for the AgInP_2_S_6_ crystal with optimized geometry as mentioned above are presented in Fig. 3. Constructed diagrams allow to conclude that the investigated crystal is indirect band gap semiconductor with the gap edges localized at the *H* and *Γ* high symmetry points (see Fig. 3b). The calculated value of the indirect energy gap *E*^calc^_gi_ ∼ 1.072 eV is significantly smaller than available experimental estimation of the optical gap which is equal to 2.43 eV.^50^ In general, this is consistent with the DFT methodology which systematically underestimates the value of the energy band gap of semiconductors. However, such essential discrepancy needs to be explained. The experimental measurements^50^ have been carried out for AgInP_2_S_6_ powder samples at room temperature by diffuse light reflection method taking into consideration only direct transitions contribution. According to presented theoretical results, the studied AgInP_2_S_6_ crystal is an indirect-gap material, moreover the lowest direct transition (*Γ*_5_ → *Γ*_1_) is forbidden by symmetry for each polarization of an incident light (see Table 3). Additionally, as it can be seen in Fig. 3b, the lowest energy level of the conduction band is formed by an energetically isolated couple of branches which give the fairly low contribution to reflection coefficient at room temperature.^50^

**Fig. 4:**
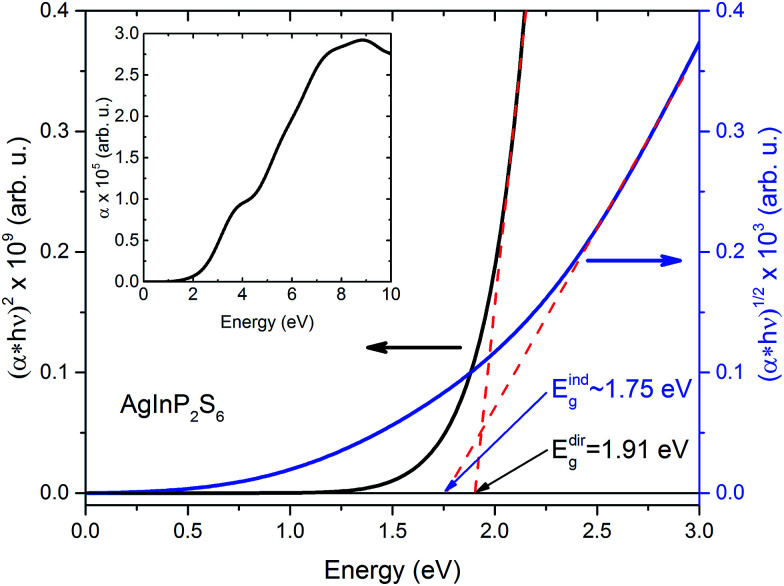
Calculated energy dependence of light absorption coefficient *α* (insertion) of the AgInP_2_S_6_ crystal and (*α* × *hν*)^2^, and (*α* × *hν*)^1/2^ curves in low energy region.

The extra validation of reliability of our results can be found by comparison of calculated (on the basis of band structure Fig. 3) direct and indirect optical gap (see Fig. 4) and experimental results.^50^ The Fig. 4 presents the calculated energy dependence of the light absorption coefficient *α* (see insertion), the (*α* × *hν*)^2^ and (*α* × *hν*)^1/2^. Based on the presented data and the simple parabolic band model the rough estimations for indirect and direct optical gaps are equal to *E*^ind^_g_ ∼ 1.75 eV and *E*^dir^_g_ = 1.91 eV, respectively. The indirect gap is defined by order of magnitude of the optical phonon energy in the *H* point (∼±0.01 eV). These values obviously are in qualitative and better quantitative agreement with results of.^50^

Additionally, in semiconducting alloys, the variations of components composition strongly affects the band gap. Ferreira *et al.* has shown that with temperature increasing of the PbSnTe the energy gap also increases.^51^ The bandgap variation with temperature measured for the CsSnI_3_ crystals shows the same uncommon temperature dependence.^52^ In present work the energy gap was calculated at the 0 K temperature and it seems that the same uncommon temperature dependence is observed.

From the fundamental solid state point of view, the calculated dispersion of the band structure is principal to calculate the effective masses of charge carriers. In present work the effective mass of electrons (m^*^_e_) was evaluated from the curvature (energy derivatives) of the bottom of conduction band in *Γ* point of the BZ and the effective mass of holes (m^*^_h_) was calculated analysing the curvature of the top of valence band in *H* point of the BZ. The diagonal elements of the effective mass tensor for electrons and holes were calculated following the equation:5
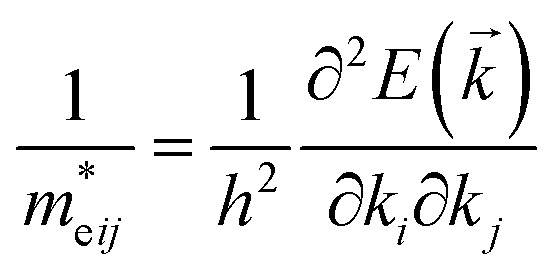


In consequence, the effective mass of electrons and holes was determined by fitting the conduction and valence band, respectively, to a parabolic function. The values of reduced effective masses of charge carriers in the AgInP_2_S_6_ crystal are as follows: 
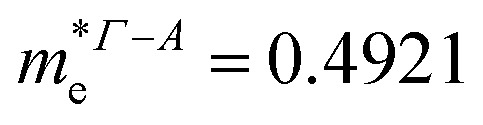
; 
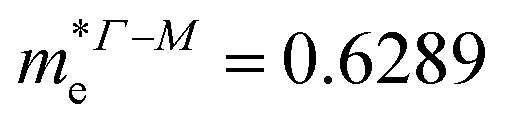
; 
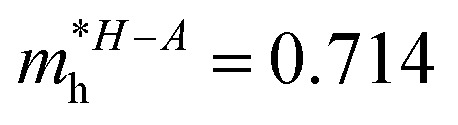
; 
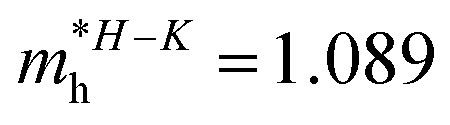
. Using these data the ratio of *m*_‖_/*m*_⊥_ = 1.28 for electrons and ratio of *m*_‖_/*m*_⊥_ = 1.53 for holes were obtained. The relatively low values of reported electrons effective masses suggest that the mobility of charges in investigated AgInP_2_S_6_ single crystal should be relatively high. Dziaugys *et al.*^4^ measured that the conductivity of AgInP_2_S_6_ crystals is ionic (Ag^+^ ions) and is equal to 10^−7^ S m^−1^ at room temperature. It is in agreement to the high value of the effective mass of holes because in ionic semiconductors the mobility of holes is very low.

**Fig. 5:**
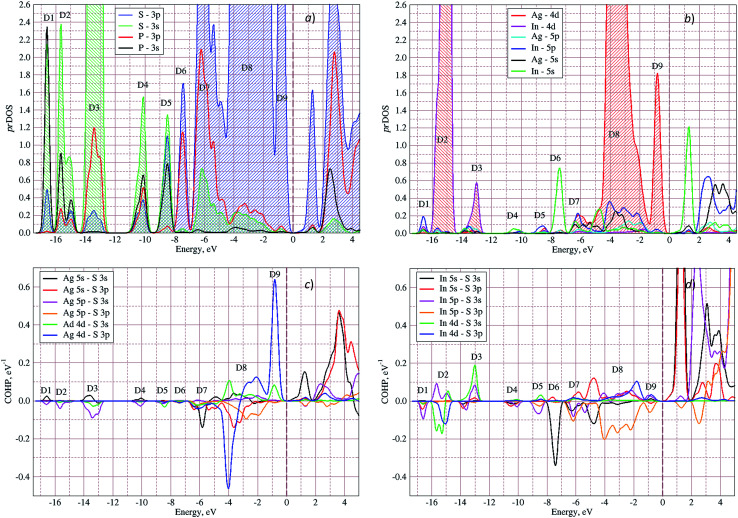
Projected densities of states (prDOS) for anion complexes [P_2_S_6_] (a) and cations (b) as well as crystal orbital Hamilton population (COHP)^53,54^ of Ag–S (c) and In–S (d) orbitals overlapping in AgInP_2_S_6_ crystal calculated by GGA/PBE-D methodology.

In Fig. 5 the calculated projected density of states (prDOS) for [P_2_S_6_] anion complexes and cation atoms are presented. Analyzing Fig. 3 and 5 one may say that the valence band can be separated into nine subbands denoted by D1…D9 (see Fig. 5). The energy band structure can be analyzed in the framework of elementary energy bands concept^47^ and, in consequence, the nature of the mentioned subbands may be explained.

Wave functions analysis of symmetry transformation properties in all high-symmetry points of the BZ defined for the AgInP_2_S_6_ crystal can designate all valence and set of the lowest conduction states. Corresponding sequences of irreps in high symmetry points *Γ*(0, 0, 0) and 
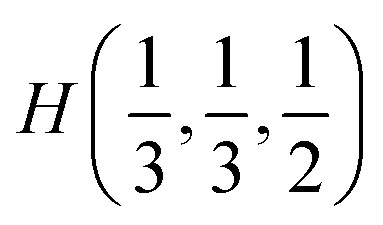
 defined as the band structure extremes are as follows:

in *Γ*(0, 0, 0) point:

(*Γ*_1_*Γ*_2_) (*Γ*_3_*Γ*_4_*Γ*_6_*Γ*_5_*Γ*_6_*Γ*_5_*Γ*_1_*Γ*_2_) (*Γ*_5_*Γ*_6_*Γ*_5_*Γ*_6_) (*Γ*_1_*Γ*_2_) (*Γ*_3_*Γ*_4_) (*Γ*_1_*Γ*_2_) (*Γ*_6_*Γ*_5_*Γ*_6_*Γ*_5_*Γ*_2_*Γ*_1_) (*Γ*_6_*Γ*_3_*Γ*_5_*Γ*_1_*Γ*_6_*Γ*_5_*Γ*_2_*Γ*_4_*Γ*_1_*Γ*_5_*Γ*_3_*Γ*_6_*Γ*_4_*Γ*_2_*Γ*_5_*Γ*_6_*Γ*_5_*Γ*_6_) (*Γ*_6_*Γ*_5_) ↓ (*Γ*_1_*Γ*_2_) (*Γ*_3_*Γ*_4_*Γ*_6_*Γ*_4_*Γ*_1_*Γ*_3_*Γ*_5_*Γ*_2_)…

in 
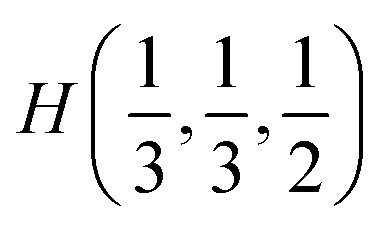
 point:

(*H*_3_) (*H*_3_*H*_3_*H*_3_*H*_1_*H*_2_*H*_3_*H*_3_) (*H*_1_*H*_2_*H*_3_*H*_2_*H*_1_*H*_3_) (*H*_3_) (*H*_3_) (*H*_1_*H*_2_) (*H*_3_*H*_1_*H*_1_*H*_3_*H*_3_*H*_2_*H*_2_) (*H*_3_*H*_1_*H*_2_*H*_3_*H*_3_*H*_1_*H*_3_*H*_1_*H*_3_*H*_2_*H*_1_*H*_2_*H*_3_*H*_3_*H*_2_*H*_3_*H*_3_*H*_3_) (*H*_1_*H*_2_*H*_3_) *↓* (*H*_1_*H*_2_) (*H*_3_*H*_3_*H*_3_*H*_1_*H*_3_*H*_2_)…

Also, the symmetry of the wave functions in all other high symmetry points of the Brillouin zone was determined (see Table 2). These band symmetry designators combined into sets correspond to subbands denoted by D1…D9 (see Fig. 5). As a result, the decomposition of the whole valence band into elementary energy bands was obtained:

D1: *Γ*_1_ + *Γ*_2_ − *M*_1_ + *M*_2_ − *L*_1_ − *A*_3_ − *K*_3_ − *H*_3_ → 
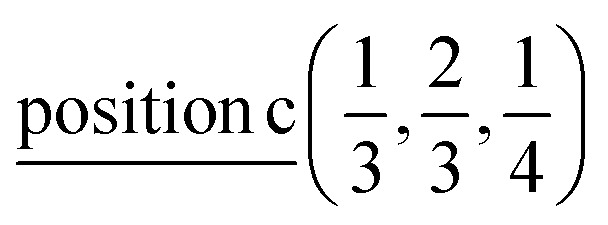
 or 
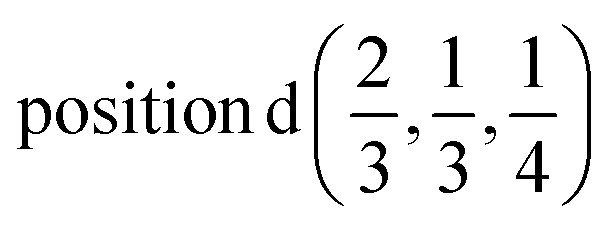
;

D2: *Γ*_3_ + *Γ*_4_ − *M*_3_ + *M*_4_ − *L*_1_ − *A*_3_ − *K*_3_ − *H*_3_ → 
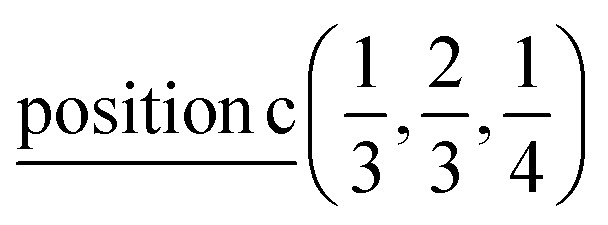
 or 
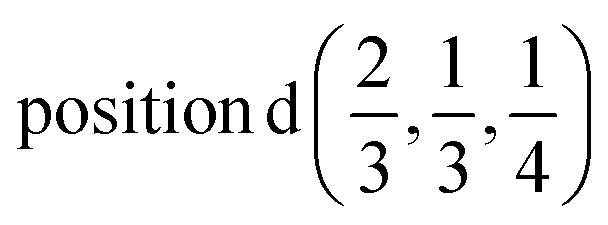
;

*Γ*_1_ + *Γ*_2_ − *M*_1_ + *M*_2_ − *L*_1_ − *A*_3_ − *K*_3_ − *H*_3_ → 
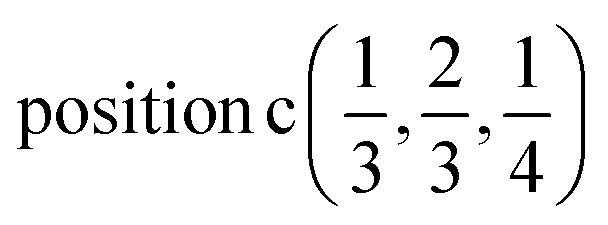
 or 
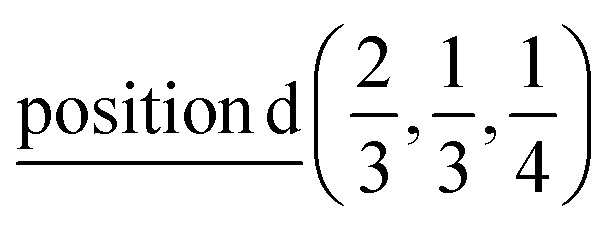
;

*Γ*_5_ + *Γ*_6_ − *M*_1_ + *M*_2_ + *M*_3_ + *M*_4_ − 2*L*_1_ − {*A*_1_ ⊕ *A*_2_} − 2*K*_3_ − 2*H*_3_ → 
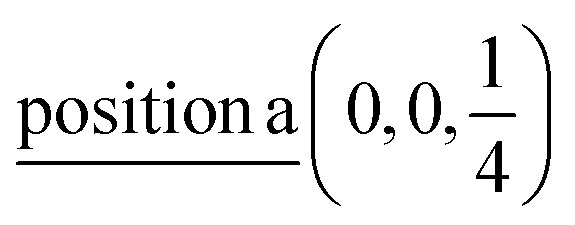
;

*Γ*_5_ + *Γ*_6_ − *M*_1_ + *M*_2_ + *M*_3_ + *M*_4_ − 2*L*_1_ − {*A*_1_ ⊕ *A*_2_} − *K*_1_ + *K*_2_ + *K*_3_ − *H*_1_ + *H*_2_ + *H*_3_ → 
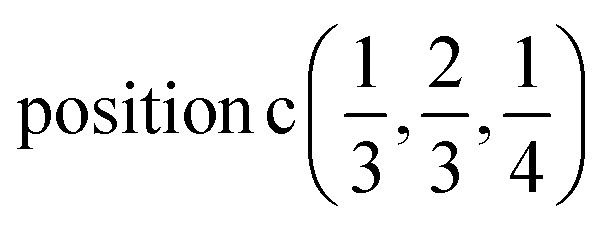
 or 
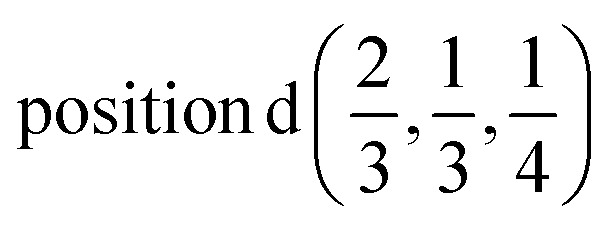
 or 
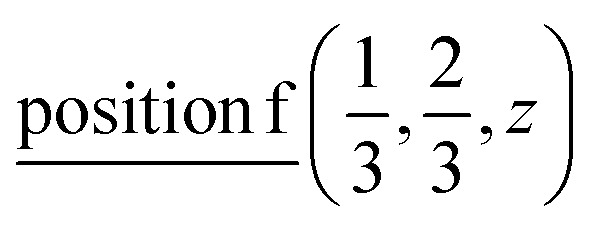
;

D3: 2(*Γ*_5_ + *Γ*_6_) − 2(*M*_1_ + *M*_2_ + *M*_3_ + *M*_4_) − 2*L*_1_ − 2{*A*_1_ ⊕ *A*_2_} − 2(*K*_1_ + *K*_2_ + *K*_3_) − 2(*H*_1_ + *H*_2_ + *H*_3_) → 
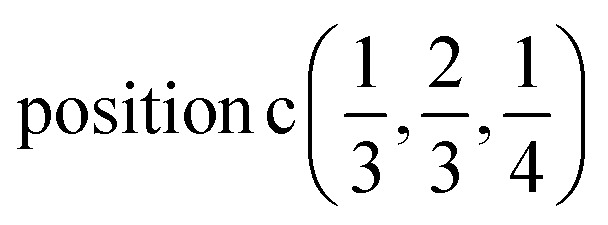
 or 
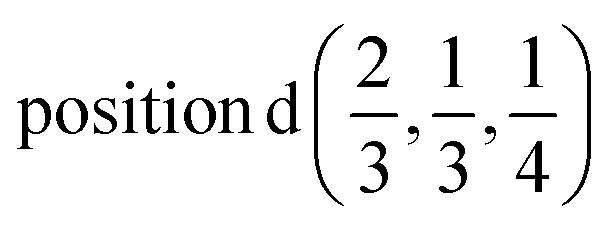
 or/and 
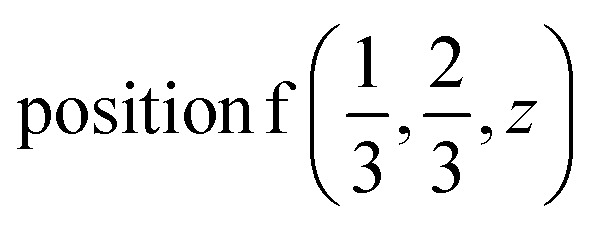
;

D4: *Γ*_1_ + *Γ*_2_ − *M*_1_ + *M*_2_ − *L*_1_ − *A*_3_ − *K*_3_ − *H*_3_ → 
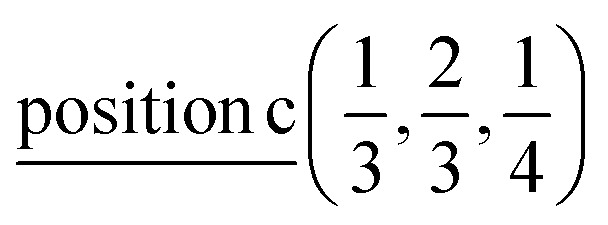
 or 
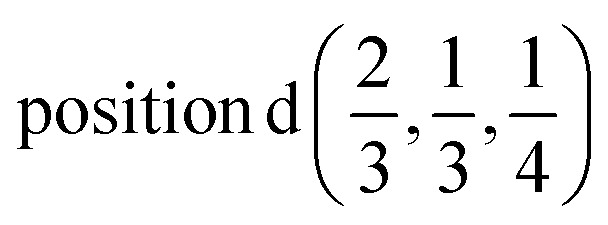
;

D5: *Γ*_3_ + *Γ*_4_ − *M*_3_ + *M*_4_ − *L*_1_ − *A*_3_ − *K*_3_ − *H*_3_ → 
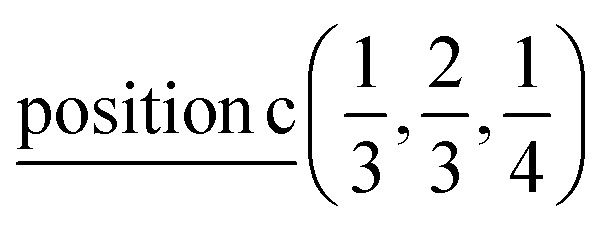
 or 
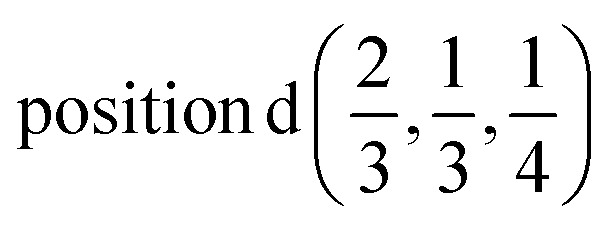
;

D6: *Γ*_1_ + *Γ*_2_ − *M*_1_ + *M*_2_ − *L*_1_ − *A*_3_ − *K*_1_ + *K*_2_ − *H*_1_ + *H*_2_ → 
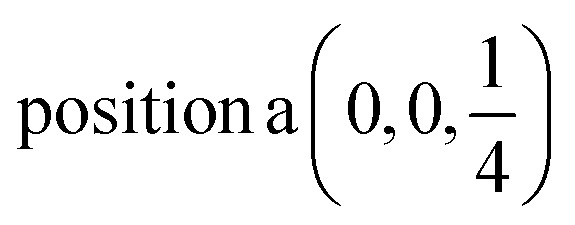
;

D7: *Γ*_1_ + *Γ*_2_ − *M*_1_ + *M*_2_ − *L*_1_ − *A*_3_ − *K*_3_ − *H*_3_ → 
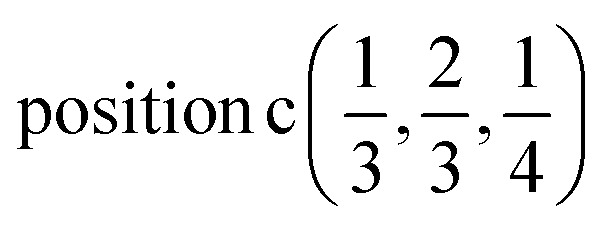
 or 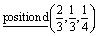;

2(*Γ*_5_ + *Γ*_6_) − 2(*M*_1_ + *M*_2_ + *M*_3_ + *M*_4_) − 4*L*_1_ − 2{*A*_1_ ⊕ *A*_2_} − 2(*K*_1_ + *K*_2_ + *K*_3_) − 2(*H*_1_ + *H*_2_ + *H*_3_) → 
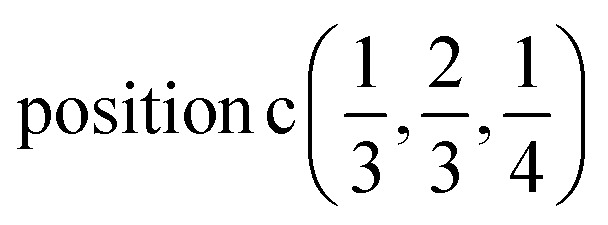
 or 
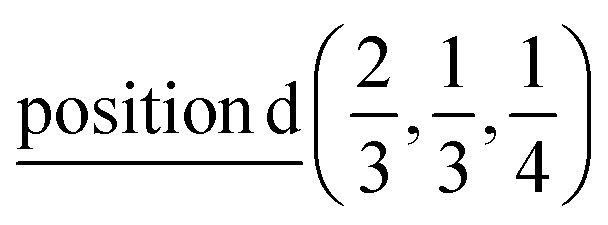
 or/and 
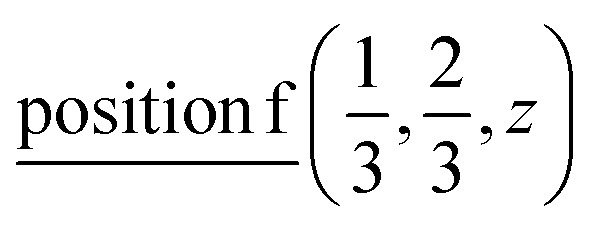
;

D8: 2(*Γ*_1_ + *Γ*_2_ + *Γ*_3_ + *Γ*_4_ + 2*Γ*_5_ + 2*Γ*_6_) − 6(*M*_1_ + *M*_2_ + *M*_3_ + *M*_4_) − 12*L*_1_ − 4({*A*_1_ ⊕ *A*_2_} + *A*_3_) − 4(*K*_1_ + *K*_2_ + 2*K*_3_) − 4(*H*_1_ + *H*_2_ + 2*H*_3_) → 
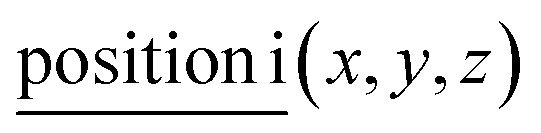
;

*Γ*_5_ + *Γ*_6_ − *M*_1_ + *M*_2_ + *M*_3_ + *M*_4_ − 2*L*_1_ − {*A*_1_ ⊕ *A*_2_} − *K*_1_ + *K*_2_ + *K*_3_ − *H*_1_ + *H*_2_ + *H*_3_ → 
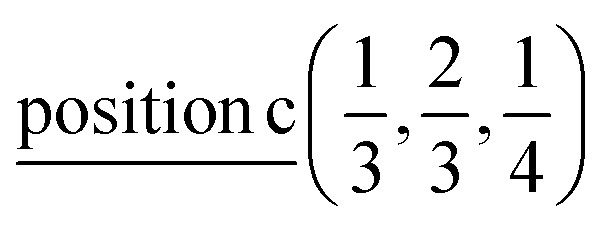
 or 
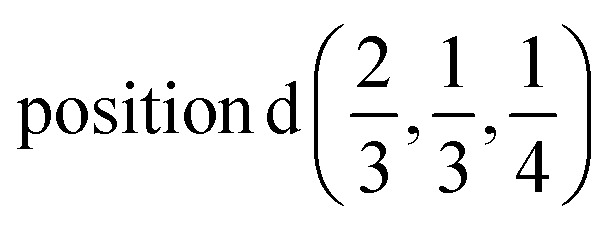
 or 
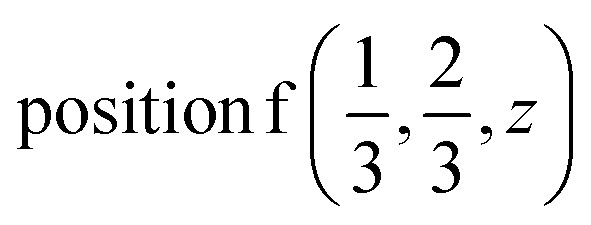
;

D9: *Γ*_5_ + *Γ*_6_ − *M*_1_ + *M*_2_ + *M*_3_ + *M*_4_ − 2*L*_1_ − {*A*_1_ ⊕ *A*_2_} − *K*_1_ + *K*_2_ + *K*_3_ − *H*_1_ + *H*_2_ + *H*_3_ → 
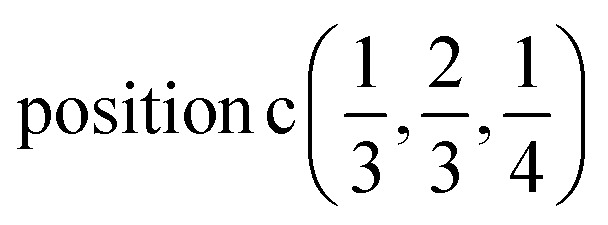
 or 
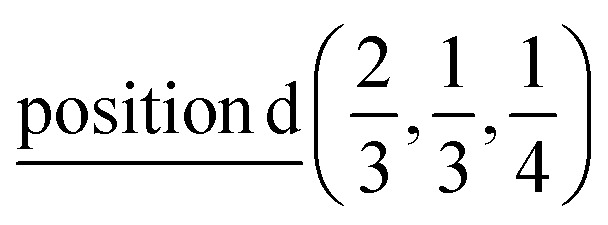
 or 
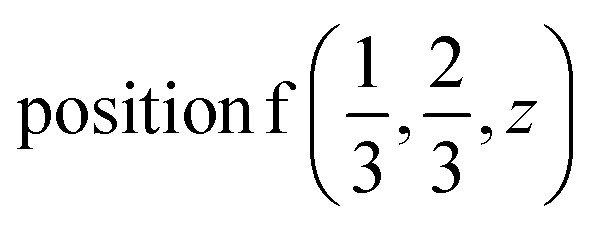
.

Above presented underlined positions are those whose contribution is dominant, as it was shown in calculated prDOS (see Fig. 5).

According to electronic configuration of atoms, the total number of occupied energy states forming the valence band is equal to 70 in each *k*-point. Analyzing the distribution of energy bands and the densities of states (pDOS, prDOS) one may conclude that the width of the valence band is 16 eV and is formed by nine distinguished bundles of branches with different genesis (see Fig. 3 and 5).

The lowest energy states (from −16 eV to −15.5 eV), labeled as D1, are built up by 3s orbitals of P and S atoms (see Fig. 5). Next valence subband D2 located from −15.5 eV up to −14 eV has an essential contribution of localized 4d electrons of indium, which are rather chemically inert in this crystal. This subband has the contribution of 3s and 3p orbitals of P and S atoms as well. In the energy region from −13.5 eV to −12.5 eV, the subband D3 formed by overlapping of S 3s, P 3s, and S 3p states and admixture of In 4d orbitals is placed. Next three couples of energy bands combined into three subbands D4, D5 and D6 are located from ∼−11 to −6.5 eV in energy scale. These bands, except the D6, are formed by 3s and 3p orbitals of S and P atoms mixed in different proportions. The D6 subband also contains the strong contribution of In 5s states. These slightly split doublets are the prominent evidence of the layered structure of the investigated crystal and represent above-mentioned Davydov's splitting,^45^ typical for crystals with translationally nonequivalent complexes in the unit cell.

**Fig. 6:**
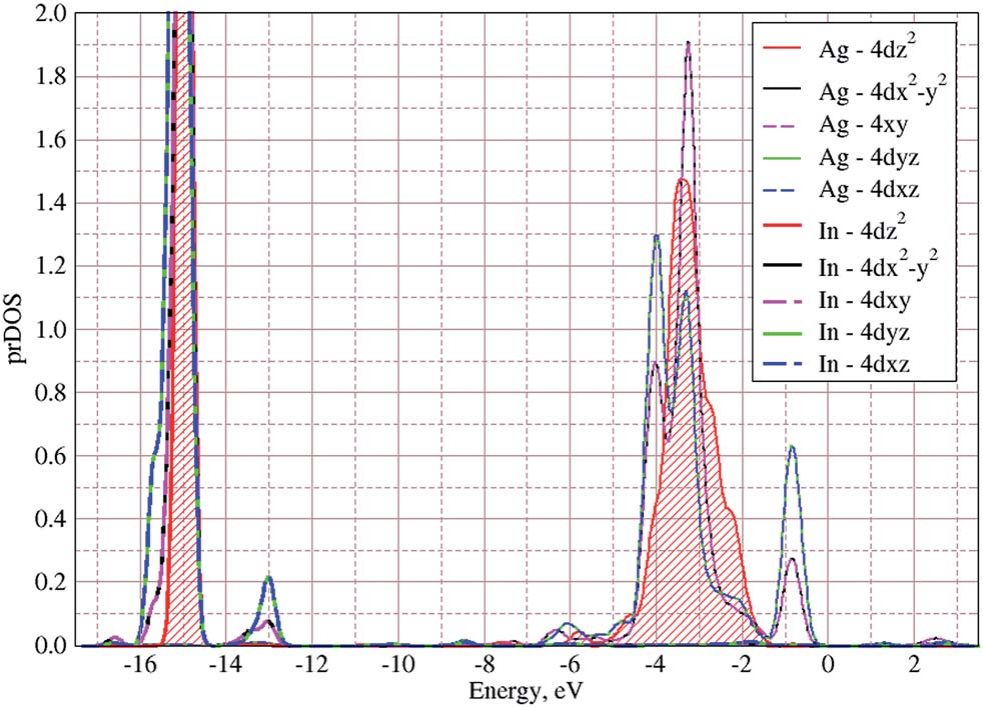
Calculated projected densities of states of d-orbitals of cations in the AgInP_2_S_6_ crystal.

The valence band branches, defined as D7 and located between −6.5 and −4 eV arises mainly from overlapping of the S 3p, P 3p, and S 3s orbitals with admixing of In 5p and 5s as well as Ag 4d and 5s states. In this energy range, the essential mixing of [P_2_S_6_] molecular orbitals with different cation orbitals (s and p for In, and s and d for Ag) take place. Both, the broadest D8 and rather narrow D9 subbands spread in energy from −4 to −1 eV and from −1 to 0 eV, respectively, are originated from 4d-states of Ag atoms and 3p-orbitals of sulfur with noticeable contribution of the P 3p and P 3s states (mainly in the D8 subband). Also, in the considered energy range (D8) one can find In 5p, Ag 5s, In 5s, and Ag 5p orbitals contribution (in order of magnitude decrease) which also indicate the complicated mixing of [P_2_S_6_]' molecular orbitals and electronic states of both metal cations. The topmost Ag 4d states are split into two separated ranges. The same situation is seen for the deep 4d states of indium. Such splitting appears due to the partial lifting of a degeneracy of atomic d-orbitals in the local crystal field. It is in agreement with the site symmetry of the In and Ag atoms located in the center of sulfur polyhedrons. It should be noted, that for ML_*n*_-type molecules (M – transition metal, L – ligands) the d-orbitals split in accordance with the scheme (t_2g_ + e_g_) for octahedral coordination or (e_g_ + t_2g_) for tetragonal coordination.^55^ However in considered structure with *D*_3d_ site symmetry the d-states combined into two higher isoenergetic pairs as (E′_g_ (d_*xy*_, d_*x*^2^−*y*^2^_) and E′′_g_ (d_*xz*_, d_*yz*_)) and one lower state with A_1g_ (d_*z*^2^_) symmetry (see Fig. 6). The interaction of metallic cation states with molecular orbitals of the whole [P_2_S_6_] anion complexes leads to mixing of the s and d states of Ag, as well as the s and p states of In (whose d orbitals are lying much lower in energy). Mentioned interaction is weaker than in pure ML_*n*_-molecules but involves almost all states and significantly raises the covalence of M–[P_2_S_6_] bonding.

The bottom of the conduction band is mainly formed by the In 5s and S 3p orbitals. As one can easily see from Fig. 5 and 6, the roles of d orbitals of two cations of Ag and In are completely distinct. It seems also important to mention that s and d orbitals of In atoms are significantly separated in energy (∼16 eV), while s and d orbitals of Ag atoms are separated by only ∼3 eV gap. Therefore, the d electrons of the In atoms form more chemically inert (no overlapping) and localized charge distribution, while self-overlapping Ag orbitals make them more labile and facilitate their delocalization as well as indirect exchange with neighboring molecular complexes. These features of cationic electrons redistribution in the AgInP_2_S_6_ crystal can be illustrated by maps of squared modulus of wave functions distribution in the plane (110).

**Fig. 7:**
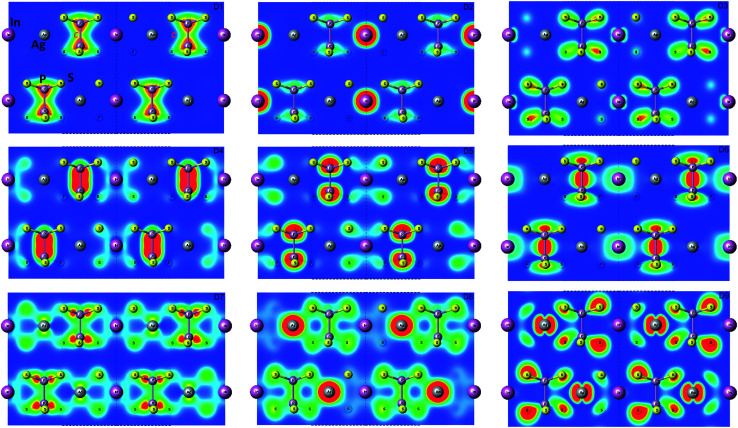
Maps of the valence energy states formed by D1…D9 subbands calculated by GGA/PBE-D methodology for AgInP_2_S_6_ crystal in *Γ* point. The visualization was done in the (110) plane.

It visualizes the charge distributions within atom layer packages (see Fig. 7). In addition, to confirm of the obtained results, the Mulliken charge population was calculated using GGA/PBE-D approach. Obtained results for s, p, d orbitals of In, Ag, P, S atoms are collected in Table 6. The presented results exhibit the cationic nature of the Ag and In atoms. It proved the localization of the In electrons and dispersed character of the Ag electrons.

**Table 6:** Mulliken charges in AgInP_2_S_6_ crystal calculated by the GGA/PBE-D method

	s	p	d	Total	Charge
Ag	0.50	0.30	9.98	10.78	0.22
In	0.94	1.27	10.0	12.21	0.79
P	1.49	2.96	—	4.45	0.55
S	1.89	4.47	—	6.35	−0.35

Discussing a stability of the AgInP_2_S_6_ seems right to compare the studied materials with a structure of other representatives of this materials family. In this case the CuInP_2_S(Se)_6_ crystal was chosen. It is well known that instability of Cu-contained crystals, successfully explained by the second-order Jahn–Teller effect (SOJT),^56^ can destabilize the closed-shell ions such as Cu^1+^, In^3+^ or Ag^1+^.

Main factor, which affect the SOJT effect realization in these crystals is the covalency of bonds and possibility of hybridization of the s and d orbitals of cation.^57^ Electronic configuration of the Cu atom, unlike the configuration of Ag or In atoms (discussed above) is characterised by the extremely close appearance of 3d- and 4s-orbitals, which are rather shallow on the energy scale. The d-orbitals of both considered atoms (Ag and Cu) can hybridize with molecular orbitals of [P_2_S_6_] anion complexes,^58^ but while Ag 4d states spread in energy in rather wide range (∼−7 to 0 eV) and the 3d states of Cu are more localized in energy (∼−5 to 0 eV).

Thus, the s–d hybridization is more efficient in the AgInP_2_S_6_ crystal than in paraelectric phase of the Cu-containing isostructures. Situation changes at Cu displacement towards trigonal coordination on ferrielectric phase transition when symmetry lowering allows more effective mixing of the s and d states of Cu stabilizing the system and making distort crystalline structure energetically preferable (this results will be published thereafter). Also, we can conclude that distinguish in cations electronic structure lead to lowering the covalence in Cu–[P_2_S(Se)_6_] bonding in comparison with Ag–[P_2_S_6_] ones. It follows from the set of experimental facts. In particular, the difference between sum of ionic radii for Cu and S ions (*r*_ion_(Cu) = 0.73 Å, *r*_ion_(S) = 1.84 Å (ref. 59)) and calculated length of Cu–S bonds (∼2.54 Å) is significantly less than the one for Ag and S ions (*r*_ion_(Ag) = 1.15 Å, *r*_ion_(S) = 1.84 Å (ref. 59)), which is about of ∼0.2 Å, that also indicates the considerable covalent contribution in Ag–S bonding. Above estimations as well as experimental values of the energy gaps (*E*_g_ = 2.43 eV for AgInP_2_S_6_,^50^ and *E*_g_ = 2.65 eV for CuInP_2_S_6_ (in paraelectric phase)^60^) also indicates the lowering of covalence on Ag to Cu changing. This tendency also reflected in vibration spectrum of above-mentioned crystals. Namely, the comparison of the lowest optical vibration frequencies of CuInP_2_S_6_ and AgInP_2_S_6_ demonstrate decrease from 25 cm^−1^ to 21 cm^−1^ (ref. 61) and corresponding squared frequency lowers to about 20% while the atomic mass growths in about 40%. This also indicates the growth of Ag–S bonds rigidity in comparison to Cu–S ones. Thus, we can state that according to our numerical modeling the AgInP_2_S_6_ crystal should be stable in contrast to isostructural Cu-containing compounds according to known experimental facts. Concerning the role of the second cation (In^3+^) in considered structures it is worth to highlight that the main contribution in chemical bonding are made by its s- and p-orbitals, while relatively low lying d-states are almost inert. Being more delocalized the In 5s- and 5p-electrons strongly mix with molecular orbitals of the anion complexes and make their contribution in almost each subband of the energy band spectrum. The In^3+^ ion plays here the role of covalently (stable) bonded electron donors. It reflects in larger charge transfer from its s- and p-orbitals (5s^0.94^5p^1.27^) in comparison to the Ag ion (5s^0.5^5p^0.3^).

### 3.3. Vibrational and elastic properties

**Fig. 8:**
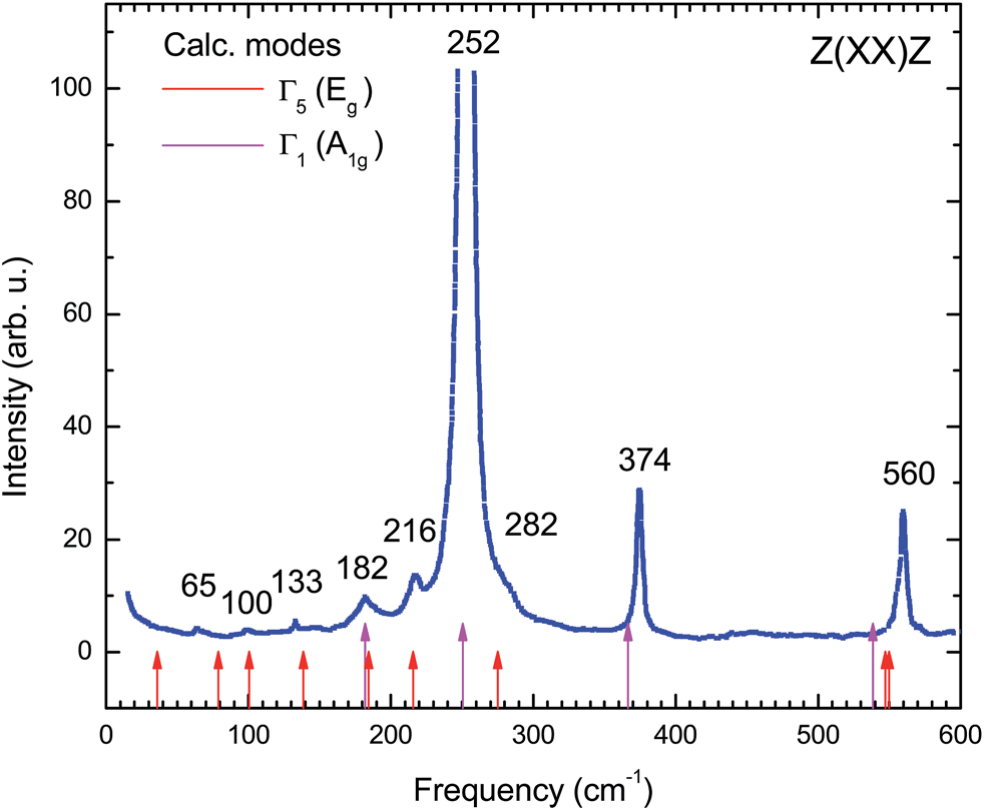
Raman scattering spectrum of AgInP_2_S_6_ crystal measured experimentally in *Z*(*XX*)*Z* geometry at room temperature.

In the present work also the dynamics of the AgInP_2_S_6_ crystal lattice was investigated and reported. The phonon frequency of the AgInP_2_S_6_ crystal was measured by polarized Raman spectroscopy. The experiment was performed in *Z*(*XX*)*Z* geometry at room temperature with 514.5 nm Ar laser excitation lines. In the high-frequency region the spectra were satisfactorily fitted to Lorentzian profiles but in the low-frequency range, they were fitted by Voight profiles (Lorentzian convoluted with a Gaussian of appropriate width). Obtained results are depicted in the Fig. 8.

To determine the nature of the experimental Raman scattering spectra they were compared to the eigenfrequencies of phonon modes calculated by GGA/PBE-D methodology (see Table 7). The calculated frequencies are also depicted in Fig. 8 by red and pink arrows. The agreement between experimental and theoretically obtained data is very good. It proves that the GGA/PBE-D methodology optimize well the structure of the AgInP_2_S_6_ crystal.

**Table 7:** Vibration modes of AgInP_2_S_6_ lattice calculated by GGA/PBE-D methodology and their comparison to experimentally measured Raman frequencies

Mode N	Frequency (cm^−1^)	Irrep.	Raman active	Raman *Z*(*XX*)*Z* [exp.]
1, 2	0.022501	E_u_ (*x*, *y*)	N	
3	0.013105	A_2u_ (*z*)	N	
4	20.926421	A_2g_ (*J*_*z*_)	N	
5	26.107787	A_2u_ (*z*)	N	
6, 7	35.979029	E_g_ (*xz*, *yz*)	Y	—
8	61.671083	A_2g_ (*J*_*z*_)	N	
9, 10	75.514009	E_u_ (*x*, *y*)	N	
11, 12	78.935074	E_g_ (*xz*, *yz*)	Y	65
13, 14	93.954836	E_u_ (*x*, *y*)	N	
15, 16	100.369898	E_g_ (*xz*, *yz*)	Y	100
17	117.278994	A_2u_ (*z*)	N	
18	127.060009	A_2g_ (*J*_*z*_)	N	
19, 20	135.102694	E_u_ (*x*, *y*)	N	
21, 22	138.545071	E_g_ (*xz*, *yz*)	Y	133
23	179.280872	A_2g_ (*J*_*z*_)	N	
24	179.576487	A_2u_ (*z*)	N	
25	180.786519	A_1u_ (*y*(3*x*^2^ − *y*^2^))	N	
26	182.083987	A_1g_ (*z*^2^)	Y	—
27, 28	184.492954	E_g_ (*xz*, *yz*)	Y	182
29, 30	185.042316	E_u_ (*x*, *y*)	N	
31, 32	215.374008	E_u_ (*x*, *y*)	N	
33, 34	215.601394	E_g_ (*xz*, *yz*)	Y	216
35	244.040140	A_1u_ (*y*(3*x*^2^ − *y*^2^))	N	
36	250.638375	A_1g_ (*z*^2^)	Y	—
37, 38	254.658638	E_u_ (*x*, *y*)	N	
39	258.888355	E_g_ (*xz*, *yz*)	Y	252
41, 42	272.692033	E_u_ (*x*, *y*)	N	
43, 44	275.028018	E_g_ (*xz*, *yz*)	Y	282
45	306.988799	A_2u_ (*z*)	N	
46	314.703934	A_2g_ (*J*_*z*_)	N	
47	366.465808	A_1g_ (*z*^2^)	Y	374
48	367.398277	A_1u_ (*y*(3*x*^2^ − *y*^2^))	N	
49	446.468206	A_2u_ (*z*)	N	
50	448.103233	A_2g_ (*J*_*z*_)	N	
51	538.402358	A_1g_ (*z*^2^)	Y	—
52	539.244632	A_1u_ (*y*(3*x*^2^ − *y*^2^))	N	
53, 54	547.301483	E_g_ (*xz*, *yz*)	Y	—
55, 56	548.380855	E_u_ (*x*, *y*)	N	
57, 58	549.779358	E_g_ (*xz*, *yz*)	Y	560
59, 60	550.875484	E_u_ (*x*, *y*)	N	

**Fig. 9:**
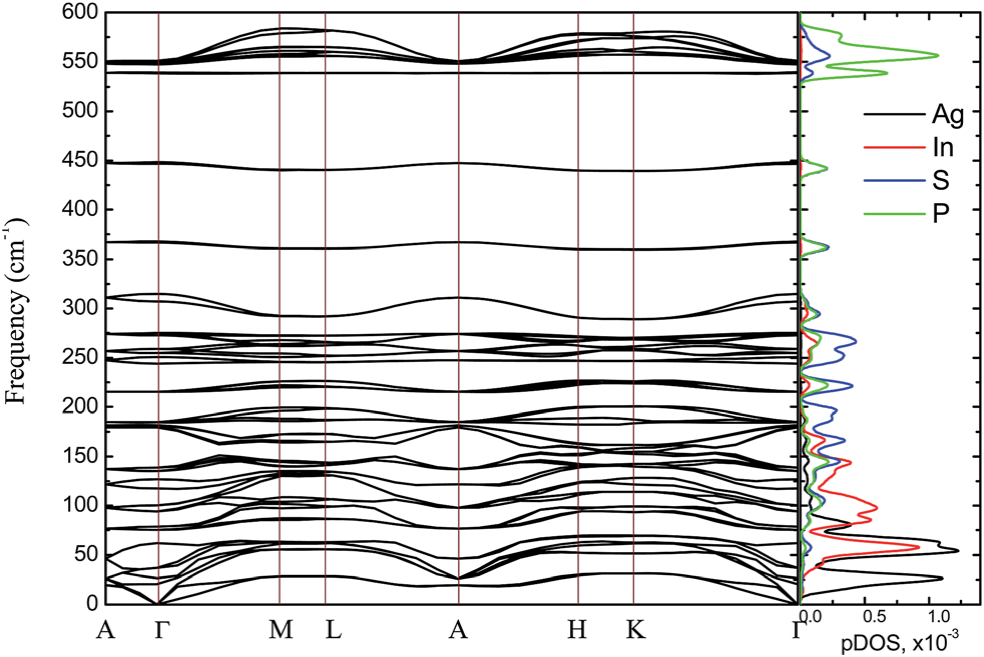
Phonon dispersion curves and phonons partial DOS of calculated by GGA/PBE-D method for AgInP_2_S_6_ crystal along the symmetry directions of the BZ.

The bulk phonon dispersions along several symmetry lines together with the corresponding phonon density of states are presented in Fig. 9. Analyzing the corresponding atoms contribution into vibrational modes the frequencies may be divided into four regions: the lowest-energy region below 35 cm^−1^, first middle region (35–175 cm^−1^), second middle region (175–400 cm^−1^) and the high-energy excitation region above 450 cm^−1^. The low-frequency range mainly consists of Ag atom vibrations. The Raman modes in this region are largely affected by the vibration of metal atoms. No Raman peak could be resolved below 50 cm^−1^ in AgInP_2_S_6_ while the peak around 25 cm^−1^ is observed in the spectrum of the CuInP_2_S_6_.^59^ The first middle region consists mostly of the In, S and P atom vibrations.

The second middle region includes only vibrations of the (P_2_S_6_)^4−^ anion complexes. It is characteristic for all hexachalcogenohypodiphosphates.^62^ In the high-frequency region, vibrations of phosphorus atoms forming P–S bonds are seen. The same results are observed for another chalcogenide crystals.^24,61^

The theoretical results presented in Table 7 indicate that there is no peak allowed by symmetry located around 450 cm^−1^ for the AgInP_2_S_6_ crystal but in our work (see Fig. 8) as well as in the work^6^ it has been detected. A presence of the mentioned peak can be an evidence of slight imperfection of investigated AgInP_2_S_6_ crystals. Wang and co-authors^6^ had attributed this peak to the stretch vibration of the P–P bond.

The elastic constants of AgInP_2_S_6_ crystal were also calculated using the GGA/PBE-D methodology. Complete set of these results is collected in Table 8. No reports on the elastic properties of the AgInP_2_S_6_ crystal have been found so far. Therefore, comparison of these results with experimentally or theoretical obtained data is not possible. The experimental data that can be used to validate the results presented in Table 8 are reported in the work of Samulionis and coauthors.^63^ The ultrasonic sound velocity measured there for the CuInP_2_S_6_ crystal along the *c*-axis is equal to 4450 m s^−1^ at 295 K. This velocity increases with decreasing temperature.

**Table 8:** The elastic modulus *B* and coefficients of elastic stiffness *C*_*ij*_ calculated by GGA/PBE-D method for AgInP_2_S_6_ crystal. All values are in GPa units except for the non-dimensional Poisson ratios *ε*_*j*_ (*i*, *j* = *x*, *y*, *z*)

Property	Value
*C*_11_	108.92750 ± 4.897
*C*_33_	39.95850 ± 9.673
*C*_44_	18.55615 ± 1.647
*C*_12_	35.81315 ± 2.981
*C*_13_	17.46785 ± 3.575
*C*_15_	5.15943 ± 0.354
Bulk modulus *B*	33.42266 ± 4.567
Young's modulus *E*_*x*_, *E*_*y*_	91.07583
Young's modulus *E*_*z*_	35.74233
*ε*_*xy*_ = *ε*_*yx*_	0.2965
*ε*_*xz*_ = *ε*_*yz*_	0.3075
*ε*_*zx*_ = *ε*_*zy*_	0.1207

In this case, the sound velocity for AgInP_2_S_6_ crystal was calculated using Christoffel equations^64^ and obtained results were compared to data measured for the Cu(In,Cr)P_2_S_6_ crystal.^26,63^

The calculated elastic stiffness constants *C*_*iklj*_ were used to construct the Christoffel matrix *Γ*_*ij*_:6
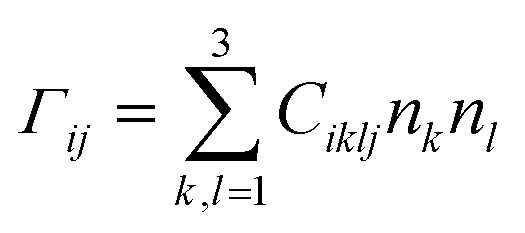
here *n*_*i*_ = cos(*k⃑*, *a⃑*_*i*_) define directional cosine defining the propagation direction of the sound wave.

The system of linear eqn (6) can be solved when the corresponding secular equation meets dependency:

7 |*Γ*_*ij*_ − *ρv*^2^*δ*_*ij*_| = 0

**Fig. 10:**
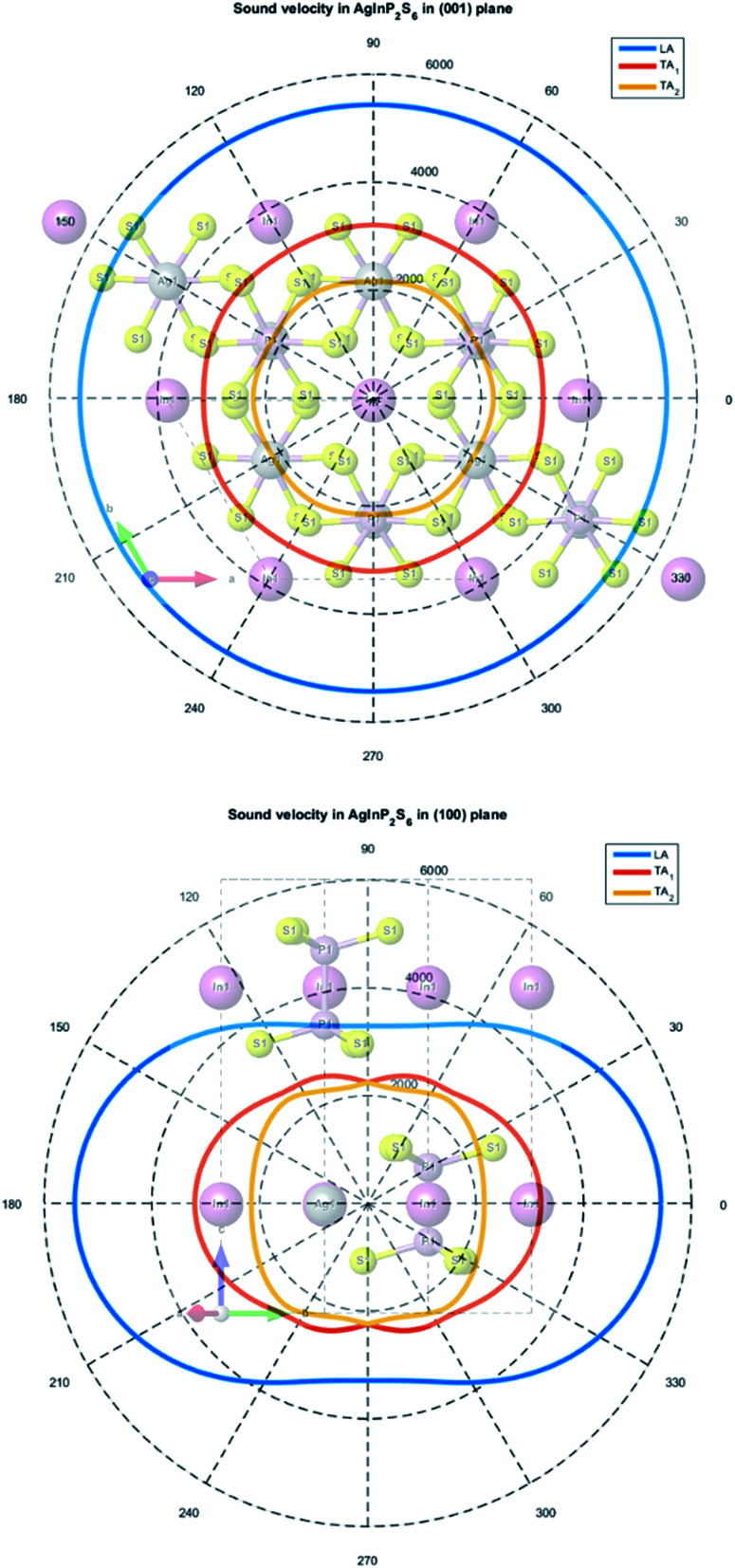
The calculated sound velocity indicatrices in the plane of the atomic layer (001) (top panel) and in the perpendicular plane (100) (bottom panel).

A solution of the secular eqn (7) allows to obtain three values of sound velocities corresponding to one longitudinal (LA) and two transverses (TA_1_, TA_2_) sound waves. The sound propagation velocity in (001) and (100) plane is presented in Fig. 10.

The maximum and minimum values of sound velocity in the plane of the atomic layer (001) and in the perpendicular plane (100) are collected in Table 9. The obtained results are in good agreement with the value measured for the CuInP_2_S_6_ and CuCrP_2_S_6_ crystals.^63,65^ It indirectly confirms that the elastic constants of the AgInP_2_S_6_ crystal were calculated properly using GGA/PBE-D methodology. The acoustic wave propagates in the (001) plane with the same velocity in all directions. It means that the AgInP_2_S_6_ crystal in (001) plane is isotropic. Perpendicularly to this plane the mentioned crystal is anisotropic. The ratio *V*_‖_/*V*_⊥_ = 1.65 can serve as a mechanical anisotropy magnitude indicator. Also the ratio of electron effective masses *m*_‖_/*m*_⊥_ = 1.28 confirm the anisotropy character of the AgInP_2_S_6_ crystal.

**Table 9:** Sound velocities in AgInP_2_S_6_ crystal calculated by GGA/PBE-D method using Christoffel' equations

	(100) plane		(001) plane	
	*V*_max_, m s^−1^	*V*_min_, m s^−1^	*V*_max_, m s^−1^	*V*_min_, m s^−1^
LA	5430.2	3288.9	5437.5	5430.2
TA_1_	3204.4	2241.7	3204.4	3145.8
TA_2_	2429.7	2156.7	2223.4	2156.7

Despite absence in literature, the experimental results on sound velocity measurements for the AgInP_2_S_6_ crystal as mentioned above, can estimate these parameters by comparison to known values for the similar crystals as the CuInP_2_S_6_ and CuCrP_2_S_6_. According to ref. 63 sound velocity in [001] direction is equal to *v*_0_ = 4460 ± 50 m s^−1^ for CuInP_2_S_6_ and *v*_0_ = 4160 ± 50 m s^−1^ for CuCrP_2_S_6_, at low temperature (polar phase), and is equal to *v*_0_ = 3875 ± 50 m s^−1^ and *v*_0_ = 3975 ± 50 m s^−1^ at high temperature (centrosymmetric phase). Our results obtained for centrosymmetric AgInP_2_S_6_ crystal (*v*_0_ = 3288 m s^−1^) are close enough to above listed experimental values for related structures.

## 4 Conclusions

In the present work, the first principles calculations of physical properties of the chalcogenide AgInP_2_S_6_ crystal was performed. First of all the crystal structure was optimized applying the GGA/PBE-D methodology. Then the electronic, vibrational and elastic constants were also calculated using the mentioned DFT functional. Comparing the theoretically obtained structural data with the experimental results was shown that for layered chalcogenide crystals the DFT-D approach should be used. The detailed structural information defined by DFT-D approach give opportunity to explain chemical bonding character of the AgInP_2_S_6_ crystal.

The formation of subbands in the energy band spectrum of the layered crystal was analyzed. In this case the reason of the AgInP_2_S_6_ crystal stability in comparison with other (almost) isostructural representatives of the considered materials family (*e.g.* CuInP_2_S_6_ or CuInP_2_Se_6_ crystals) can be elucidated. Having the electronic structure calculated for the AgInP_2_S_6_ crystal one can assume that stability of this compound in comparison with Cu-containing structures, in particular, is based on a rather big covalence of the Ag–[P_2_S_6_] bonds and therefore more rigid Ag–S polyhedra (this is an important factor for realization or absence of the SOJT effect mechanism, which is often employed in analysis of phase transitions in this type of crystals).

For more detailed understanding physical properties of the AgInP_2_S_6_ crystal, the lattice dynamics was investigated within the GGA/PBE-D approach. The phonon dispersion curves and partial density of the AgInP_2_S_6_ crystal phonon states were calculated. Theoretically obtained results were compared with experimental data. Satisfactory agreement between calculated and experimental vibrational modes was achieved.

For the first time, the elastic modulus, coefficients of elastic stiffness of AgInP_2_S_6_ crystal and Poisson ratios were calculated in presented work. These data were used to calculate the sound velocity in the plane of the (001) atomic layer and in the perpendicular plane (100). Obtained results are in good agreement with the experimental results.

The presented work confirm that the electronic and structural properties of the layered chalcogenide structure can be characterized theoretically applying the DFT methodology at the GGA level of approximation adding the van der Waals correction. It seems to be reasonable for the layered structure characterized by van der Waals interlayer gap.

## Conflicts of interest

There are no conflicts to declare.

## Supplementary Material
